# Conjugative delivery of toxin genes *ccdB* and *kil* confers synergistic killing of bacterial recipients

**DOI:** 10.1128/jb.00168-25

**Published:** 2025-07-03

**Authors:** Yang Grace Li, Daniel Haeusser, William Margolin, Peter J. Christie

**Affiliations:** 1 Department of Microbiology and Molecular Genetics, McGovern Medical School, UTHealth Houston 12339 , Houston, Texas, USA; National Institutes of Health, Bethesda, Maryland, USA

**Keywords:** conjugation, horizontal DNA transfer, mobile genetic elements, antibiotic resistance, type IV secretion, antimicrobials

## Abstract

The bacterial type IV secretion systems (T4SS) are medically problematic for their roles in the dissemination of mobile genetic elements or effector proteins, but they also have great potential for new antimicrobial therapies. Recent studies have deployed the T4SS subfamily of conjugation systems to deliver gene editing CRISPR/Cas systems to disrupt drug resistance genes or kill targeted bacterial recipients. However, the therapeutic potential of conjugative CRISPR/Cas delivery is compromised by mutations or host repair systems that diminish the efficiency with which CRISPR/Cas induces double-strand breaks in new transconjugants. Here, we compared the efficiencies of conjugation-based killing systems based on the delivery of CRISPR-Cas9 elements or toxin genes encoding the bacteriophage lambda Kil peptide or the F plasmid-encoded CcdB. *Escherichia coli* equipped with one of two efficient conjugation systems, pKM101 (IncN) or F (IncF), served as donors to mobilize plasmids carrying the cognate *oriT* sequence and one or more toxic elements. Overall, toxin gene delivery proved significantly more effective than CRISPR-Cas9 in killing of transconjugant population, but the highest levels of growth suppression of both *E. coli* and *Klebsiella pneumoniae* recipients were achieved by a combination of CRISPR-Cas9 plus one or two toxin genes. By contrast, capsule production conferred no or very slight protective effects on plasmid acquisition and killing of either species. We propose that the conjugative co-transfer of two or more toxic elements with distinct mechanisms of action has strong potential for growth suppression of targeted species in environmental or clinical settings.

**IMPORTANCE:**

The prevalence of antibiotic resistance emphasizes the need for alternative antimicrobial intervention strategies. We engineered *Escherichia coli* for conjugative transmission of plasmids encoding CRISPR-Cas9 elements or genes encoding the cell division inhibitor Kil or gyrase poisoner CcdB. Delivery of toxin genes more effectively suppressed the growth of *E. coli* recipients than CRISPR-Cas9, but the combinatorial delivery of CRISPR-Cas9 and a toxin gene or two toxin genes elicited the strongest killing effects. Capsule production by *E. coli* or *Klebsiella pneumoniae* recipient cells had no or little protective effect on plasmid acquisition or growth suppression. Our findings suggest that probiotic donor strains equipped for conjugative delivery of two or more toxic elements may prove effective as an alternative or adjunct to traditional antimicrobials.

## INTRODUCTION

The increasing prevalence of antibiotic resistance emphasizes an urgent need for innovative antibacterial strategies. Promising therapeutic alternatives include the deployment of lytic bacteriophages (1, 2), nanoparticles loaded with toxic elements (3–6), or probiotic bacteria with natural secretion systems engineered to deliver toxic elements to targeted pathogens (7–10). Among the latter, the type IV secretion systems (T4SSs) have strong potential for interbacterial killing systems. They are widely dispersed among many bacterial species, where they function in the intercellular translocation of effector proteins or mobile genetic elements (MGEs) (11–13). And while the large T4SS subfamily of conjugation systems is medically problematic for their roles in dissemination of MGEs with their cargoes of virulence and antibiotic resistance determinants (14–16), mobile elements can be deleted of their cargo genes and engineered to carry CRISPR/Cas (clustered regularly interspaced short palindromic repeats/Cas nuclease) systems (17–20). The conjugative transfer of MGEs encoding CRISPR/Cas systems has various translational applications, ranging from the modification of targeted bacterial genomes, curing of plasmids or antibiotic resistance genes, or killing of targeted species through generation of double-stranded breaks (DSBs) (21–25).

Recently, we developed an interbacterial killing system that relied on *Escherichia coli* donor cells equipped with highly efficient conjugative plasmids for delivery of CRISPR-Cas9 elements with guide RNA sequences targeting a *chl^R^* gene in recipient cells. We deployed well-established conjugation systems, including F (IncF) and pKM101 (IncN), that transfer at high frequencies under dense cell growth conditions. The F system also conjugates efficiently in lower-density aqueous conditions, likely due to the elaboration of long, flexible F pili that dynamically extend and retract to draw distant recipient cells into close donor cell contact to promote the formation of mating junctions (26, 27). While the pKM101 system is less efficient in aqueous conditions, it has the advantage over the F system for its capacity to deliver DNA cargoes to a broader range of Enterobacterial species than F (28–31). Both F and pKM101 can also efficiently deliver mobilizable plasmids to recipient cells. Mobilizable plasmids characteristically lack *tra* genes encoding T4SSs for self-transmission, but harbor origin-of-transfer (*oriT*) sequences and associated mobilization (*mob*) genes required for processing the plasmids for transfer. In our experimental system, we equipped *E. coli* donors with F or pKM101 and mobilizable plasmids bearing CRISPR-Cas9 elements and the *oriT* sequences from F or pKM101; we designated these as pCrispr plasmids. We showed that the *E. coli* donors efficiently mobilized pCrispr plasmids, resulting in an impressive 10^3^-fold killing of new transconjugant cells. However, ~10^3^–10^5^ transconjugants also escaped CRISPR-Cas9-mediated killing (18). Such high “escape” frequencies have also been reported by other groups evaluating the efficacy of conjugation-mediated CRISPR-Cas9 delivery systems (17, 19, 20, 32–34). As reported with traditional antibiotic therapies, therefore, the potency of CRISPR/Cas systems for genome editing or targeted killing can be significantly mitigated by intrinsic or acquired resistance among targeted cell populations (25).

In this study, we evaluated the relative effectiveness of conjugation-mediated delivery of plasmids encoding CRISPR-Cas9 systems vs protein toxins for growth suppression of *E. coli* and *Klebsiella pneumonia* recipients. We report that mobilization of plasmids encoding the F-encoded gyrase inhibitor CcdB (35) or bacteriophage lambda Kil that inhibits cell division (36, 37) confers appreciably greater killing of transconjugant cell populations than delivery of plasmids harboring a CRISPR-Cas9 system. Strikingly, delivery of plasmids carrying combinations of CRISPR-Cas9 and *kil* or *ccdB* and *kil* could achieve nearly complete elimination of transconjugant populations and as much as ~10^5^-fold killing of the entire recipient population. Our findings suggest that combinatorial therapies consisting of conjugation-mediated delivery of multiple toxic elements constitute a promising therapeutic alternative to antibiotics, especially for problematic multidrug-resistant infections.

## MATERIALS AND METHODS

### Bacterial growth conditions

*E. coli* and *K. pneumoniae* strains used in this study are described in Table 1. Both species were grown at 37°C with shaking in Lysogeny Broth (LB) or on solid LB agar. Strains or plasmids were maintained by selection with the following antibiotics with final concentrations in parentheses: carbenicillin (100 µg/mL), spectinomycin (100 µg/mL), chloramphenicol (20 µg/mL), kanamycin (100 µg/mL), tetracycline (20 µg/mL), gentamycin (20 µg/mL), and rifampicin (100 µg/mL).

**TABLE 1:** Strains used in this study

Strain	Genotype or description	Source
*Escherichia coli*		
MC4100	F^-^ [*araD139*]*_B/r_*Δ(*argF-lac*)*U169*λ^-^ e14^-^ *ﬂhD5301*Δ(*fruK-yeiR*)*725*(*fruA25*) *relA1 rpsL150 rbsR22*Δ(*ﬁmB-ﬁmE*)*632*(::*IS1*) *deoC*	(38)
MC4100-Rif	Rif^R^; MC4100 mutated to rifampin resistance	(18)
MC4100-Chl	Chl^R^; MC4100 with chromosomally-integrated *chl^R^*	(18)
AA116	Rif^R^ Chl^R^; MC4100-Chl mutated to rifampin resistance	(39)
YGLS9	Kan^R^; MC4100 with chromosomally integrated P*chl^R^*::*ccdA kan^R^*	This study
WM1657	Kan^R^; MG1655 Δ*lacU169 ftsA_R286W_* (ftsA*) *zipA*::*kan^R^*	(40)
DH5α	F^-^ φ80d*lacZ*Δ*M15* Δ(*lacZYA-argF*)*U169 deoR recA1 endA1 hsdR17*(*rK*^-^*mK*^+^) *phoA supE44* λ^-^*thi-1 gyrA96 relA1*	Gibco-BRL/Invitrogen
HD-143	MC4100 *rcs* λatt (P*rprA-lacZ*) Δ*yfaS rcsC137*	Gift from Anna Konovalova
YGLS11	Rif^R^; HD-143	This study
*Klebsiella pneumoniae*		
KPPR1S	Rif^R^; ATCC 43816 derivative	(41)
VK248	Rif^R^; KPPR1S Δ*rcsB*	(41)

### Strain construction

*E. coli* MC4100 strain YGLS9 carries a chromosomal copy of *ccdA* constitutively expressed from the upstream *chl^R^* promoter (P*_chlR_*). It was constructed by PCR amplification of *ccdA* and *kan^R^* using pYGL613 as a template. The PCR product was then inserted into the *chl^R^* gene of MC4100-Chl by recombineering using pKD46 and selection for recombinants on LB plates containing kanamycin (100 µg/mL) (42, 43). Recombinants were verified by PCR amplification across the *chl^R^* gene and by sequencing the amplicons. The resulting strain constitutively expresses *ccdA* from the *chl^R^* promoter.

### Plasmid constructions

Plasmids and oligonucleotides used in this study are described in Tables 2 and 3. *pOX38 derivatives:* pOX38Δ*ccdAB* is a *ccdAB* deletion mutant of pOX38::Tet. It was constructed by PCR amplification of *spc^R^* using pBAD101 as a template and inserting the PCR product into the *ccdAB* operon of pOX38::Tet by recombineering using pKD46 (43). Recombinants were selected on LB plates containing spectinomycin (100 µg/mL). Recombinants were verified by PCR amplification across the *ccdAB* operon and by sequencing the amplicons. pYGL687 is pOX38::Tet carrying P_tac_::*ccdB*. It was constructed by PCR amplification of P_tac_::*ccdB* and the linked *gnt^R^* gene using pYGL681 as a template and inserting the PCR product into *tet^R^* of pOX38::Tet by recombineering. Recombinants were selected on LB plates containing gentamycin (20 µg/mL). Tetracycline-sensitive recombinants were verified by PCR amplification across *tet^R^* and sequencing the amplicon. pYGL686 is the isogenic toxin minus control of pYGL687. It was constructed by PCR amplification of the P_tac_ promoter and *gnt^R^* using pYGL680 as a template and inserting the PCR product into *tet^R^* of pOX38::Tet by recombineering. Recombinants were selected on LB plates containing gentamycin (20 µg/mL). Tetracycline-sensitive recombinants were verified by PCR amplification across *tet^R^* and sequencing the amplicons.

**TABLE 2:** Plasmids used in this study

Plasmid	Description	Source
Cloning vectors		
pBAD24	Crb^R^; ColE1 with P_BAD_ promoter	(44)
pRR48_MCSpKG_	Crb^R^; ColE1 with P_tac_ promoter	(36)
pBAD101	Spc^R^; pSC101 with P_BAD_ promoter	(45)
pML122	Gnt^R^; mobilizable RSF1010 derivative	(46)
pDSG-null	Kan^R^; p15A producing a β-barrel of intimin autotransporter but no passenger domain	(47)
*E. coli* expression plasmids		
pKM101	Spc^R^; IncN conjugative plasmid	(48)
pOX38::Tet	Tet^R^; Tra^+^ F plasmid derivative	(49)
FΔ*ccdAB*	Tet^R^ Spc^R^; pOX38 deleted of *ccdA ccdB*	This study
pYGL686	Gnt^R^; pYGL687 deleted from *ccdB*	This study
pYGL687	Gnt^R^; pOX38::P_tac_::*ccdB*	This study
pDH94	Crb^R^; pRR48_MCSpKG_ with P_tac_::*kil*	(36)
pYGL533	Crb^R^; pBBR with pKM101 *oriT* sequence and CRISPR-Cas9 gRNA-	(18)
pYGL535	Crb^R^; pBBR with pKM101 *oriT* sequence and CRISPR-Cas9 gRNA*_chlR_*	(18)
pYGL555	Crb^R^; pBBR with pOX38 *oriT* sequence and CRISPR-Cas9 gRNA-	(18)
pYGL557	Crb^R^; pBBR with pOX38 *oriT* sequence and CRISPR-Cas9 gRNA*_chlR_*	(18)
pYGL658	Crb^R^; pYGL533 with P_tac_ promoter	This study
pYGL665	Crb^R^; pYGL535 with P_tac_::*kil*	This study
pYGL660	Crb^R^; pYGL555 with P_tac_ promoter	This study
pYGL667	Crb^R^; pYGL557 with P_tac_::*kil*	This study
pYGL620	Crb^R^; ColE1 with P_tac_ promoter; NotI site deleted from pRR48_MCSpKG_	This study
pYGL621	Crb^R^; pYGL620 with P_tac_::*ccdB*	This study
pYGL680	Gnt^R^; ColE1 with P_tac_ promoter	This study
pYGL681	Gnt^R^; pYGL680 with P_tac_::*ccdB*	This study
pYGL634	Crb^R^; pYGL620 with pKM101 *oriT* sequence	This study
pYGL642	Crb^R^; pYGL634 with P_tac_::*ccdB*	This study
pYGL760	Crb^R^; pYGL634 with P_tac_::*kikA*	This study
pYGL635	Crb^R^; pYGL620 with pOX38 *oriT* sequence	This study
pYGL643	Crb^R^; pYGL635 with P_tac_::*ccdB*	This study
pYGL761	Crb^R^; pYGL635 with P_tac_::*kikA*	This study
pYGL630	Crb^R^; pYGL620 with pKM101 *oriT* sequence and *lacIq* deleted	This study
pYGL638	Crb^R^; pYGL630 with P_tac_::*kil*	This study
pYGL631	Crb^R^; pYGL620 with pOX38 *oriT* sequence and *lacIq* deleted	This study
pYGL639	Crb^R^; pYGL631 with P_tac_::*kil*	This study
pYGL722	Spc^R^; pSC101 with P_proDp_::*ccdA*	This study
pYGL724	Tet^R^; pSC101 with P_proDp_::*ccdA*	This study
pYGL735	Gnt^R^; pSC101 with P_proDp_::*ccdA*	This study
pYGL733	Crb^R^; pYGL634 with P_tac_::*ccdB-kil*	This study
pYGL734	Crb^R^; pYGL635 with P_tac_::*ccdB-kil*	This study
pNF02-mLychee	Chl^R^; Mini-F with P_proDp_::*mlychee*	(50)
pNF02-mLemon	Chl^R^; Mini-F with P_proDp_::*mlemon*	(50)
pYGL727	Crb^R^; pYGL635 with P_proDp_::*mlemon*	This study
pYGL728	Crb^R^; pYGL643 with P_proDp_::*mlemon*	This study
pYGL731	Crb^R^; pYGL631 with P_proDp_::*mlemon*	This study
pYGL732	Crb^R^; pYGL639 with P_proDp_::*mlemon*	This study
pYGL741	Crb^R^; pYGL734 with P_proDp_::*mlemon*	This study
pYGL612	Crb^R^; pBAD24 with P_BAD_::*ccdA*	This study
pYGL613	Kan^R^; pBAD24-Kan with P_BAD_::*ccdA*	This study
pKD46	Crb^R^; temperature-sensitive plasmid for lambda-RED recombineering in *E. coli*	(43)

**TABLE 3:** Oligonucleotides used in this study

Oligo name	Sequence (5′ to 3′)	Purpose
Cm-ccdA_F	AATGGCATCGTAAAGAACATTTTGAGGCATTTCAGTCAGTTGCTCAATGTCTGGCTCTTCTCGCTAACCAAACCGGTAAC	YGLS9
Cm-ccdA_R	TAATTCATTAAGCATTCTGCCGACATGGAAGCCATCACAGACGGCATGATGCGTAAATCAATCTAAAGTATATATGAGTAAACTTGGTCTGACAG	YGLS9
ccdAB-spec_F	TTGACAGCGACAGCTATCAGTTGCTCAAGGCATATGATGTCAATATCTCCGCTCGTTCGCCAGCCAGGACAGAAATG	pOX38Δ*ccdAB*
ccdAB-spec_R	TGGCCAGGGGGATCACCATCCGTCGCCCGGGCGTGTCAATAATATCACTCGAGCAATTATGTGCTTAGTGCATCTAACGCTTGAG	pOX38Δ*ccdAB*
pOX38-ccdB-gent_F	TACTCAGTGCCTGTTATAAGCAGCAATTAATTATGATTGATGCCTACATCCGCTTACAGACAAGCTGTGACCGTCTC	pYGL686 pYGL687
pOX38-ccdB-gent_R	CTGGCTCTGCACCTTGGTGATCAAATAATTCGATAGCTTGTCGTAATAATGAAGTTTTAAATCAATCTAAAGTATATATGAGTAAACTTGGTCTGACAG	pYGL686 pYGL687
pRRev-SA_F	GCATGTAAATGGATGTCATCCATGATGCA	pYGL620
pRRev-SA_R	CTAGTGCATCATGGATGACATCCATTTACATGCTGCA	pYGL620
NotI-pKM101-oriT_F	ATATATGCGGCCGCGTACCCTCATTTAGAATGATGTAATTTTGATGTATTTCTG	pYGL634
NotI-pKM101-oriT_R	ATATATGCGGCCGCCAGTCCTCACATTGTGCATTTCTTAAACAAAAGATT	pYGL634
NotI-pOX38-oriT_F	ATATATGCGGCCGCAATCTACCTGCATCAGTCCGCTGCC	pYGL635
NotI-pOX38-oriT_R	ATATATGCGGCCGCGCTGATATACAGGTTCACCTTAGCCATTAG	pYGL635
NotI-pRR_F	ATATATGCGGCCGCGATTCATTAAGACTCTAGAGCTCGTGATACG	pYGL634 pYGL635
NotI-pRR_R	ATATATGCGGCCGCGTGGCAACGCCAATCAGCAACGAC	pYGL634 pYGL635
SalI-pKM101-oriT_F	ATATATGTCGACGTACCCTCATTTAGAATGATGTAATTTTGATGTATTTCTG	pYGL630
SalI-pKM101-oriT_R	ATATATGTCGACCAGTCCTCACATTGTGCATTTCTTAAACAAAAG	pYGL630
oriT-OX_SalI_F	ATATATGTCGACAATCTACCTGCATCAGTCCGCTGCC	pYGL631
oriT-OX_SalI_R	ATATATGTCGACGCTGATATACAGGTTCACCTTAGCCATTAGAG	pYGL631
PstI-ccdB_F	ATATATCTGCAGAATGCAGTTTAAGGTTTACACCTATAAAAGAGAGAGC	pYGL621, pYGL642, pYGL643
SpeI-ccdB_R	ATATATACTAGTTTATATTCCCCAGAACATCAGGTTAATGGCGTTTTTG	pYGL621, pYGL642, pYGL643
PstI-kikA_F	ATATATCTGCAGCATGAAGAAACTCTTAATACCTCTGATAGCAGCTG	pYGL760
SpeI-kikA_R	ATATATACTAGTCTATAAGCGAACTTTCCCGTATTTACTTATGATCTG	pYGL760
pRR-Gent-I_F	ATTGAAAAAGGAAGAGTATGTTACGCAGCAGCAACGATGTTACGCAG	pYGL680 pYGL681
pRR-Gent-I_R	TAAACTTGGTCTGACAGTTAGGTGGCGGTACTTGGGTCGATATCAAAG	pYGL680 pYGL681
pRR-Gent-V_F	TAACTGTCAGACCAAGTTTACTCATATATACTTTAGATTG	pYGL680 pYGL681
pRR-Gent-V_R	CATACTCTTCCTTTTTCAATATTATTGAAGCATTTATCAGG	pYGL680 pYGL681
GA-pRR_F	GGGTTGCGCAGCAACCCCCCCGCTTACAGACAAGCTGTGACCGTCTC	pYGL658, pYGL665, pYGL660, pYGL667
GA-pRR-lacIq_R	TATTTGAATGTATTTAGCCCCACCACCCTGAATTGACTCTCTTCCGG	pYGL658, pYGL665, pYGL660, pYGL667
ccdB-kil_F	AAGATGTTTCGTGAAGCCGTCGACGCTTATAAAAAATGGATATTAATACTGAAACTGAGATCAAGCAAAAGCATTCACTAAACTAGTGGTACCGAGCTCCTCGAGGATC	pYGL733 pYGL734
ccdB-kil_R	TTCATCGCCAATAAAAGTGGCGATAGTGAATTTAGTCTGGATAGCCATAAGTGTTTGATCCATGGTGAATTCCTCCTTTATATTCCCCAGAACATCAGGTTAATGGCGTTTTTGATGTC	pYGL733 pYGL734
NheI-ccdA_F	ATATATGCTAGCAGGAGGAATTCACCATGAAGCAGCGTATTACAGTGACAGTTGACAG	pYGL612
HindIII-ccdA_R	ATATATAAGCTTTCACCAGTCCCTGTTCTCGTCAGCAAAAG	pYGL612
NotI-Kan_F	ATATATGCGGCCGCGAGAGCTTTGTTGTAGGTGGACCAGTTGG	pYGL613
NotI-Kan_R	ATATATGCGGCCGCCTCTGCCAGTGTTACAACCAATTAACCAATTCTG	pYGL613
NotI-Amp_F	ATATATGCGGCCGCGACAGATCGCTGAGATAGGTGCCTCAC	pYGL613
NotI-Amp_R	ATATATGCGGCCGCGCCATCCGTCAGGATGGCCTTCTG	pYGL613
pSC101-proDp-ccdA-I_F	GCTCGTATAATATATTCAGGGAGACCACAACGGTTTCCCTCTACAAATAATTTTGTTTAACTTTTACTAGAGAAAGAGGAGAAAGCTAGCATGAAGCAGCGTATTACAGTGACAGTTGAC	pYGL722
pSC101-proDp-ccdA-I_R	CGGTTTGGTTAGCGAGAAGAGCCAGTCACCAGTCCCTGTTCTCGTCAGC	pYGL722
pSC101-proDp-ccdA-V_F	CTGGCTCTTCTCGCTAACCAAACCG	pYGL722
pSC101-proDp-ccdA-V_R	GAATATATTATACGAGCCTTATGCATGCCCGTAAAGTTATCCAGCAACCACTCATAGACCTAGGGCAGCAGATAGGGACGACGTGGTGTTAGCTGTGCGAGTTCCGTGCCGGTTGTGAAG	pYGL722
pYGL722-TET-V_F	GACATTCTTGCAGGTATCTTCGAGCCAGC	pYGL724
pYGL722-TET-V_R	CAAGGTTCTGGACCAGTTGCGTGAGC	pYGL724
pYGL722-TET-I_F	GCAACTGGTCCAGAACCTTGGGCATCAAATTAAGCAGAAGGCCATCCTGAC	pYGL724
pYGL722-TET-I_R	AAGATACCTGCAAGAATGTCGTATATATGAGTAAACTTGGTCTGACAGTTAAGCACTTG	pYGL724
ccdA-gent-V_F	GTCGCTGCCGACTGGGCAATGGAG	pYGL735
ccdA-gent-V_R	GCGCCGTTACCACCGCTGCGTTC	pYGL735
ccdA-gent-I_F	CGCAGCGGTGGTAACGGCGCCATGATAATAATGGTTTCTTAGACGTCAGGTGGCAC	pYGL735
ccdA-gent-I_R	ATTGCCCAGTCGGCAGCGACGGTCTGACAGTTAGGTGGCGGTACTTGG	pYGL735
pToxin-mlemon-I_F	GAGCCATGTCGTCGTCAACGCGACCTGTAACAGAGCATTAGCGCAAG	pYGL727, pYGL728, pYGL731, pYGL732, pYGL741
pToxin-mlemon-I_R	CTCAATGCTGCTTGCTGTTCGAAGGTGAGCCAGTGTGATACTAGAGG	pYGL727, pYGL728, pYGL731, pYGL732, pYGL741
pToxin-mlemon-V_F	GAACAGCAAGCAGCATTGAGAACTTTGGAATC	pYGL727, pYGL728, pYGL731, pYGL732, pYGL741
pToxin-mlemon-V_R	CGTTGACGACGACATGGCTCGATTG	pYGL727, pYGL728, pYGL731, pYGL732, pYGL741

*Plasmids expressing ccdB and/or kil, or kikA:* pYGL620 carrying the P_tac_ promoter was derived from pRR48_MCSpKG_. pYGL621 expressing P_tac_::*ccdB* was constructed by PCR amplification of *ccdB* using pOX38 as a template, digestion of the PCR fragment with PstI and SpeI, and ligation of the resulting product with similarly digested pYGL620. pYGL680 and pYGL681 are gentamycin-resistant versions of pYGL620 and pYGL621, respectively. They were constructed by ligating PCR-amplified versions of pYGL620 or pYGL621 lacking *bla^R^* to PCR-amplified *gnt^R^* obtained from pML122 (46) by Gibson assembly (51). pYGL634 and pYGL635 are pYGL620 plasmids carrying the origin of transfer (*oriT*) sequences of pKM101 and pOX38, respectively. They were constructed by PCR amplification of pYGL620 and *oriT* sequences from pKM101 and pOX38 with compatible NotI ends and ligation. pYGL642 and pYGL643 expressing P_tac_::*ccdB* were constructed by PCR amplification of *ccdB* using pOX38 as a template, digestion of the PCR fragment with PstI and SpeI, and ligation of the resulting product with similarly digested pYGL634 and pYGL635, respectively. pYGL760 and pYGL761 expressing P_tac_::*kikA* were constructed by PCR amplification of *kikA* using pKM101 as a template, digestion of the PCR fragment with PstI and SpeI, and ligation of the resulting product with similarly digested pYGL634 and pYGL635, respectively. pYGL630 and pYGL631 are pYGL620 plasmids deleted of *lacIq* and carrying the *oriT* sequences of pKM101 and pOX38, respectively. They were constructed by PCR amplification of the *oriT* sequences using pKM101 and pOX38 as templates, digestion of the PCR fragments with SalI, and ligation of the resulting products with similarly digested pYGL620. pYGL638 and pYGL639 expressing P_tac_::*kil* were constructed by digestion of pDH94 with PstI and SpeI, and ligation of the resulting smaller fragment with similarly digested pYGL630 and pYGL631, respectively. pYGL733 and pYGL734 expressing P_tac_::*ccdB-kil* were constructed by adding the *kil* sequence on pYGL642 and pYGL643, respectively, by inverse PCR. pYGL665 is pYGL535 expressing P_tac_::*kil*. It was constructed by PCR amplification of P_tac_::*kil* using pDH94 as a template, SmaI digestion of pYGL535, and ligation of the resulting fragments by Gibson assembly. pYGL658 is the isogenic negative control of pYGL665. It was constructed by PCR amplification of the P_tac_ promoter using pYGL620 as a template, SmaI digestion of pYGL533, and ligation of the resulting fragments. pYGL667 is pYGL557 expressing P_tac_::*kil*. It was constructed by PCR amplification of P_tac_::*kil* using pDH94 as a template, SmaI digestion of pYGL557, and ligation of the resulting fragments. pYGL660 is the isogenic negative control of pYGL667. It was constructed by PCR amplification of the P_tac_ promoter using pYGL620 as a template, SmaI digestion of pYGL555, and ligation of the resulting fragments.

*Plasmids expressing ccdA:* pYGL612 expressing arabinose-inducible P_BAD_::*ccdA* was constructed by PCR amplification of *ccdA* using pOX38 as a template, digestion of the PCR fragment with NheI and HindIII, and ligation of the resulting product with similarly digested pBAD24. pYGL613 expressing P_BAD_::*ccdA* was constructed by PCR amplification of *kan^R^* using pDSG-null (equivalent to pDSG323, Addgene Plasmid #115594) (47) as a template, PCR amplification of pBAD24 with P_BAD_::*ccdA* using pYGL612 as a template, digestion of the PCR fragments with NotI, and ligation of the resulting products.

pYGL722 with constitutively expressed *ccdA* from the P_proDp_ promoter (52) was constructed by (i) PCR amplification of *ccdA* using pOX38 as a template and using pSC101-proDp-ccdA-I_F and pSC101-proDp-ccdA-I_R as primers (see primer table; pSC101-proDp-ccdA-I_F contains partial sequence of P_proDp_), (ii) PCR amplification of pSC101 using pBAD101 as a template and pSC101-proDp-ccdA-V_F and pSC101-proDp-ccdA-V_R as primers (see primer table; pSC101-proDp-ccdA-V_R contains partial sequence of P_proDp_), and (iii) ligation of the resulting fragments. pYGL724 (TetR) and pYGL735 (GntR) expressing P_proDp_::*ccdA* were constructed by PCR amplification of *tet^R^* from pOX38::Tet or *gnt^R^* from pML122, and ligation of these antibiotic resistance genes to PCR-amplified pYGL722 expressing P_proDp_::*ccdA* (but lacking the original *spc^R^* gene).

*Plasmids expressing fluorescent reporter genes:* pYGL727, pYGL728, pYGL731, pYGL732, and pYGL741 expressing P_proDp_::*mlemon* were constructed by PCR amplification of *mlemon* using pNF02-mLemon (50) as a template, PCR amplification of plasmid backbone with the toxin genes or their isogenic negative control sequences using pYGL635, pYGL643, pYGL631, pYGL639, and pYGL734 as templates, respectively, and ligation of the resulting fragments.

### Conjugation assays

*E. coli* donor and *E. coli* or *K. pneumonia* recipient strains were grown overnight with antibiotic selection, diluted 100-fold in LB broth without antibiotics, and incubated at 37°C with shaking for 2.5 h (for *E. coli–E. coli* matings) or 4 h (for *E. coli–K. pneumonia*). Donors and recipients were mixed in a 1:1 ratio, determined by colony-forming-unit (CFU) counts, unless otherwise stated. For solid-surface matings, mating mixes (30 µL) were deposited onto sterile nitrocellulose filters (0.45 µm pore size) and incubated for 2 h at 37°C on LB agar plates. For liquid matings, mating mixes (100 µL) were diluted 10-fold in LB broth. Following incubation for 1 h at 37°C, matings were terminated by vigorous vortexing (filter matings were resuspended in 1 mL LB) for 1 min and kept on ice. Donor and recipient strains carried selectable antibiotic resistance markers in their chromosomes that are distinct from each other, as well as the selectable marker carried on the transmitted plasmid. Serial dilutions of mating mixes were deposited onto LB plates containing antibiotics for quantitation of donors (D’s), recipients (R’s), or transconjugants (Tc’s). Matings were carried out at least three times in triplicate. Frequencies of plasmid transfer were reported as the number of Tc’s per donor cell (Tc’s/D). Killing efficiencies resulting from the transfer of plasmids encoding toxic elements were reported as the number of Tc’s or R’s relative to the number of Tc’s or R’s arising from the transfer of the control poriT plasmids. Three datapoints from representative experiments are presented, together with averages and standard deviations. *P*-values between indicated data sets were calculated by the homoscedastic Student’s *t*-test.

### Agar-top mating assays

Fluorescently tagged *E. coli* donor (mLemon) and recipient (mLychee) cells were grown overnight with antibiotic selection, diluted 100-fold in LB broth without antibiotics, and incubated at 37°C with shaking for 2.5 h. Donor cells were mixed in 1:1 or 100:1 ratios with recipient cells determined by CFU counts. The mixtures (20 µL) were spotted on LB agar plates containing 0.5 mM Isopropyl-β-D-thiogalactopyranoside (IPTG) and allowed to grow overnight at 37°C. Fluorescence of the mixed culture was examined using the Bio-Rad ChemiDoc MP Imaging System. Donor and recipient cells were examined by Alexa 488 and Sypro Ruby applications at auto optimal exposures, respectively.

### Growth curves

YGLS9 strains carrying constitutively expressed *ccdA* integrated into the chromosome and either pccdB or poriT (both with the F plasmid *oriT* sequence) were grown overnight with antibiotic selection and diluted 100-fold in LB broth without antibiotics. KPPR1S and isogenic variant Δ*rcsB* strains carrying either pkikA or poriT (both with the pKM101 *oriT* sequence) were grown overnight with antibiotic selection and diluted 100-fold in LB broth containing 0.5 mM IPTG without antibiotics. Cultures were incubated at 37°C with shaking overnight in the BioTek SynergyH1 microplate reader. Cell densities (OD_600_) were recorded every 15 minutes, and growth curves were plotted by Microsoft Excel.

## RESULTS

### Conjugative delivery of *kil* or *ccdB* confers more proficient killing than CRISPR-Cas9

**Fig 1:**
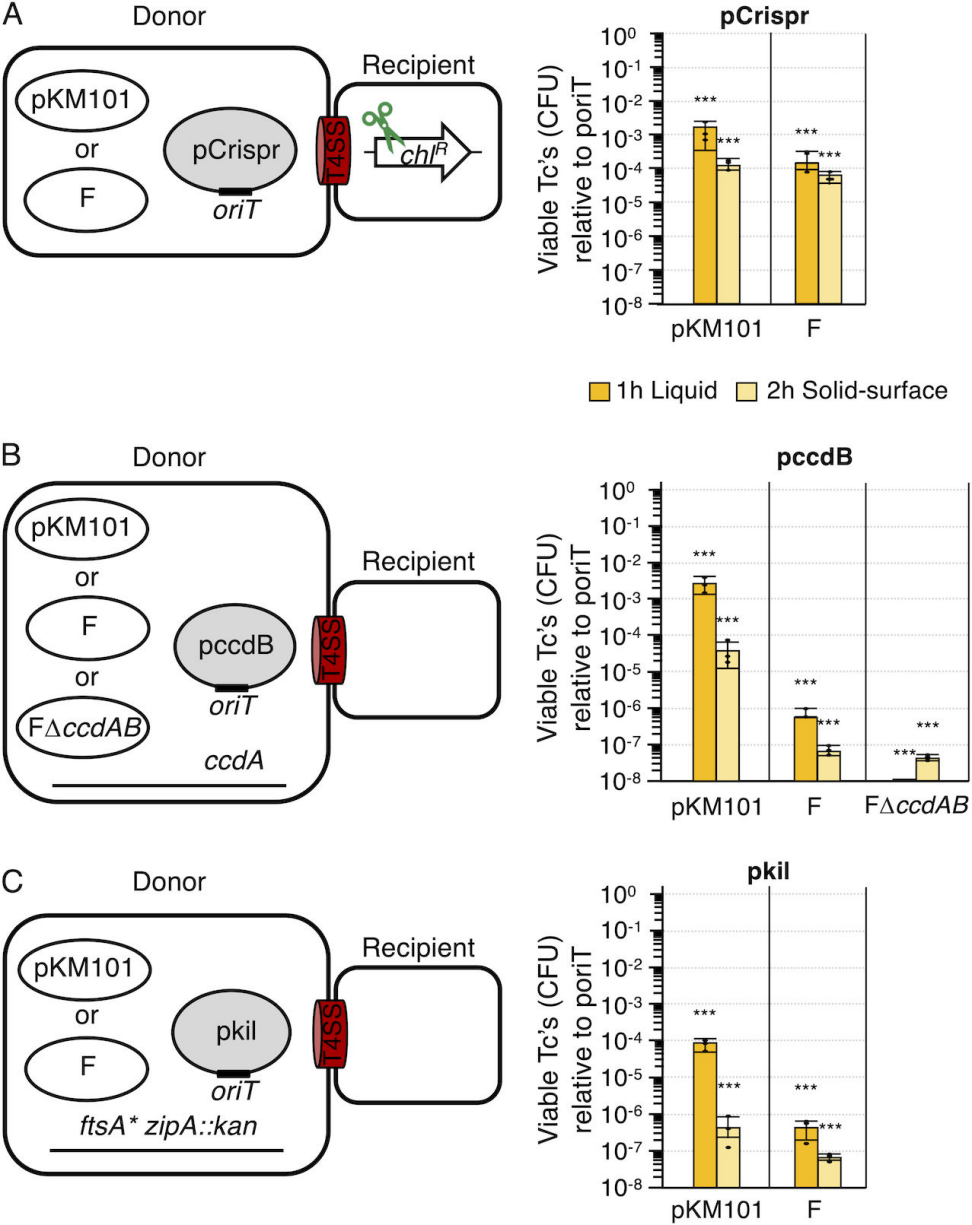
Effects of delivering plasmids encoding CRISPR-Cas9, *ccdB,* or *kil* on transconjugant viability. (**A**) Left: Schematic depicting matings between *E. coli* MC4100 donors carrying conjugative plasmids and mobilizable pCrispr and MC4100 recipients with the CRISPR-Cas9 target (*chl^R^*, scissors). Right: Transconjugant (Tc) killing presented as viable Tc’s (colony-forming units; CFUs) resulting from the transfer of pCrispr relative to the poriT vector-only plasmid (normalized to 10^0^). For this and subsequent Figure panels, the mobilizable plasmid conferring killing is in bold at the top of the panel, and the conjugative plasmids mediating transfer of the mobilizable plasmid are listed at the bottom. (**B**) Left: Schematic depicting matings between *E. coli* YGLS9 carrying conjugative plasmids and mobilizable pccdB and MC4100 recipients. Right: Transconjugant killing presented as viable Tc’s (colony-forming units; CFUs) resulting from the transfer of pccdB relative to poriT (normalized to 10^0^). (**C**) Left: Schematic depicting matings between *E. coli* WM1657 donors carrying conjugative plasmids and mobilizable pkil and *E. coli* MC4100 recipients. Right: Transconjugant killing presented as viable Tc’s resulting from the transfer of pkil relative to poriT (normalized to 10^0^). All matings were repeated at least three times in triplicate, with average transconjugant killing presented as orange (1 h liquid mating) or yellow (2 h solid surface) bars. Tc CFUs arising from a representative triplicate experiment are shown as black datapoints. Standard deviations are shown as error bars. *P*-values at the top of each bar represent comparisons with the transfer of the poriT control plasmid. ***P < 0.0001.

Consistent with our previous findings (18), F- or pKM101-mediated delivery of pCrispr plasmids to *E. coli* MC4100-Rif bearing a *chl^R^* gene elicited ~10^3^- to 10^4^-fold killing of *E. coli* transconjugants (Fig. 1A). However, an appreciable number of “escaper” cells also arose (~10^3^–10^5^), which mitigates the potency of this strategy for therapeutic intervention. This is best illustrated by quantifying the effects of CRISPR-Cas9-mediated killing on the total recipient cell population, wherein pKM101- or F-directed mobilization of pCrispr did not confer a detectable decrease in the total recipient cell population for reasons that are discussed further below (Fig. S1).

The conjugative transfer of genes encoding protein toxins has also been shown to elicit killing (25), and here we directly compared the killing efficiencies resulting from the transfer of pCrispr vs plasmids coding for toxins. We first engineered high-copy number ColE1 plasmids designated as pccdB to carry the *oriT* sequence associated with pKM101 or F along with *ccdB* (controller of cell death or division B), the product of which confers toxicity by inhibiting DNA gyrase activity. CcdB along with its CcdA antidote are the toxin and antitoxin components of a type II TA system encoded by and involved in the maintenance of F plasmids (53, 54). To counteract CcdB toxicity in *E. coli* donor cells, we engineered the *E. coli* chromosome to constitutively express *ccdA*. We confirmed that donors harboring pccdB and chromosomal *ccdA* showed no appreciable growth defects compared with isogenic donors harboring the poriT vector plasmid (Fig. S2A).

Using the pKM101-encoded T4SS (hereafter T4SS_pKM_) as the delivery system, mobilization of pccdB resulted in ~10^3^- and ~10^4^-fold killing efficiencies in liquid and solid-surface matings, respectively; these mating conditions are intended to reflect matings that occur in nature under comparatively low-density planktonic vs solid-surface biofilm or other higher-density growth conditions. Overall, T4SS_pKM_-mediated mobilization of pccdB elicited killing at levels comparable to frequencies observed with pCrispr mobilization (Fig. 1A and B). However, with the F-encoded transfer system (T4SS_F_), pccdB mobilization suppressed transconjugant growth by 10^6^- and 10^7^-fold in liquid and solid-surface matings, respectively. This translates to a ~3 log greater killing effect than conferred with T4SS_F_-directed mobilization of pCrispr (Fig. 1A and B). In these matings, the F plasmid co-transfers at equivalent high frequencies as the mobilizable plasmid (Fig. S2B). Because F encodes the CcdA antidote, we reasoned that F co-transfer might mitigate CcdB-mediated killing of transconjugants. To examine this possibility, we deleted *ccdAB* from F, resulting in FΔ*ccdAB*. Indeed, donors with FΔ*ccdAB* and pccdB more strongly suppressed transconjugant cell growth than donors harboring F and pccdB, yielding an overall >10^7^-fold reduction of the Tc population in both liquid and solid-surface matings (Fig. 1B).

Prior studies had supplied evidence that the self-transfer of conjugative plasmids engineered to carry CRISPR-Cas9 elements conferred greater killing of targeted populations than could be achieved by mobilization of smaller plasmids harboring these elements via chromosomally integrated forms of the equivalent conjugation systems (19). These findings were attributed to the capacity of self-transmissible plasmids, but not mobilizable plasmids, to reiteratively transfer through the recipient cell population. As we had shown that F and the cognate poriT harboring the F *oriT* region co-transfer at the same high frequencies to recipients (Fig. S2B), we compared the killing efficiencies conferred by donor strains equipped with F expressing P_tac_::*ccdB* or with F and the mobilizable pccdB plasmid. We observed similar levels of killing of the transconjugant population regardless of whether *ccdB* was expressed from self-transmissible F or mobilizable pccdB (Fig. S2C). Indeed, when we quantified the viable recipients recovered from the two matings, we found that mobilization of pccdB conferred slightly greater killing of the total recipient population than F expressing P_tac_::*ccdB* (Fig. S2C). We attribute these findings to the comparatively high copy number, and thus higher toxin gene dosage, of the poriT plasmid (ColE1 replicon, 30–40 copies) relative to F (1–2 copies). Also, because F mobilizes the cognate poriT plasmid (Fig. S2A) and, by extension, pccdB, at the same high frequency as observed for transfer of F itself, it is reasonable to predict that F and pccdB propagate through the recipient population as efficiently as F bearing P_tac_::*ccdB* in *cis*.

We next tested whether the conjugative transfer of a small antimicrobial peptide designated Kil elicits killing of the transconjugant population. Kil is encoded by bacteriophage λ and functions as an adjunct to λ’s main lytic program (55). Recently, λ Kil was shown to act in a ZipA-dependent manner to bind the essential division protein FtsZ and block its capacity to self-assemble into rings, resulting in cell filamentation and loss of *E. coli* cell viability (36, 37, 56). We introduced pKM101- and F-mobilizable pkil plasmids constitutively expressing *kil* in *E. coli* strain WM1657 (40), which harbors mutations (ftsA* and Δ*zipA*) that render Kil unable to block FtsZ polymerization (36). WM1657 donors additionally equipped with pKM101 and F were used to mobilize the pkil plasmids into Kil-sensitive *E. coli* MC4100-Rif (Fig. 1C). Interestingly, T4SS_pKM_-directed transfer of pkil strongly suppressed growth of transconjugants by >10^4^-fold and >10^6^-fold for liquid and solid-surface matings, respectively. This translates to ~10^1^- and 10^2^-fold greater killing efficiencies than observed for T4SS_pKM_ mobilization of pCrispr or pccdB under the comparable mating conditions (Fig. 1A through C). T4SS_F_-directed transfer of pkil also elicited significant >10^6^-fold killing of the Tc population, similar to levels achieved with T4SS_F_-mediated transfer of pccdB (Fig. 1B and C) but appreciably higher than achieved with T4SS_F_ mobilization of pCrispr (Fig. 1A and C).

### CRISPR-Cas9/*kil* or *kil*/*ccdB* co-delivery eliminates the entire transconjugant cell population

**Fig 2:**
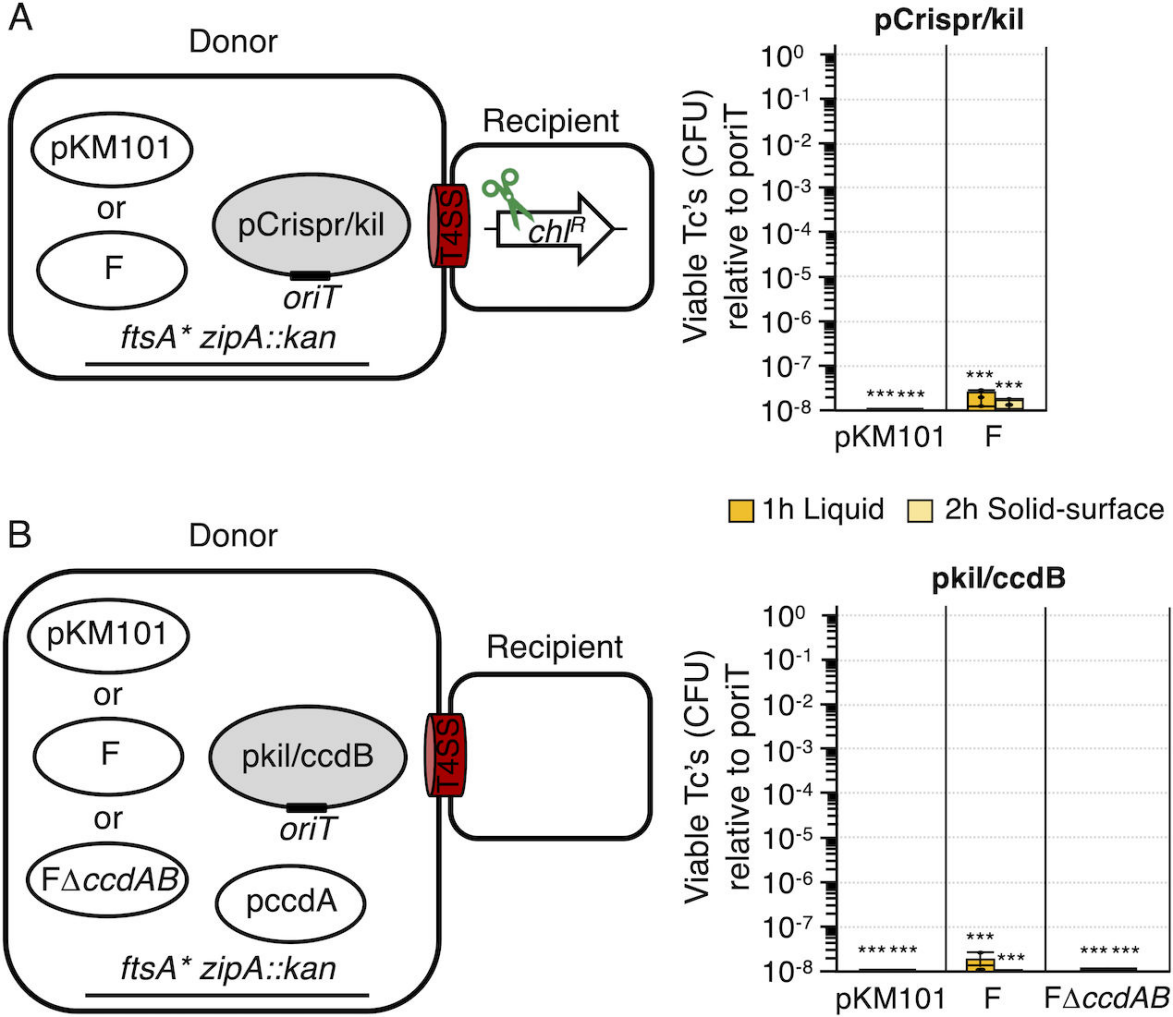
Effects of delivering plasmids encoding two toxic elements on transconjugant viability. (**A and B**) Left: Schematic depicting matings between *E. coli* WM1657 donors carrying conjugative plasmids and (**A**) pCrispr/kil or (**B**) pkil/ccdB and nontransmissible pccdA. Right: Transconjugant (Tc) killing presented as viable Tc's (colony-forming units; CFUs) resulting from the transfer of the mobilizable plasmids (listed above each panel) relative to viable Tc's resulting from transfer of the poriT vector-only plasmid (normalized to 10^0^). All matings were repeated at least three times in triplicate, with average transconjugant killing presented as orange (1 h liquid mating) or yellow (2 h solid-surface) bars. Tc CFUs arising from a representative triplicate experiment are shown as black datapoints. Standard deviations are shown as error bars. *P*-values at the top of each bar represent comparisons with transfer of the poriT control plasmid. ***P < 0.0001.

Although conjugative transfer of *ccdB* or *kil* through the T4SS_pKM_ or T4SS_F_ systems elicited 10^3^- to 10^7^-fold killing of Tc’s, variable numbers of “escaper” Tc’s also arose, likely due to acquisition of mutations that rendered these toxins ineffective (Fig. 1A through C). To combat the growing problem of antimicrobial resistance, it is now common practice to administer two or more antibiotics with distinct cellular targets to reduce the probability of resistance through spontaneous mutations. By analogy, we reasoned that conjugation-mediated transfer of two or more toxic elements would eliminate the Tc “escaper” cells. To test this prediction, we constructed two sets of mobilizable *oriT* plasmids, one carrying CRISPR-Cas9 and *kil* (pCrispr/kil) and the second carrying *kil* and *ccdB* (pkil/ccdB). WM1657 harboring the Kil-inactivating mutations along with pKM101 or F served as donors for mobilization of pCrispr/kil; isogenic strains additionally harboring a *ccdA* expression plasmid were used for mobilization of pkil/ccdB (Fig. 2A and B).

Remarkably, in many repetitions of liquid or solid-surface matings in which donors mobilized pCrispr/kil or pkil/ccdB through the T4SS_pKM_, we failed to detect growth of any Tc colonies (Fig. 2A and B). This translates to a >10^8^-fold reduction in the growth suppression of recipient cells receiving these toxin-producing plasmids. Delivery of pCrispr/kil or pkil/ccdB through the F system achieved similar ~10^8^-fold killing efficiencies in at least six repetitions of these matings, although one or two resistant transconjugants arose in a few experimental repetitions (Fig. 2A and B). However, when we substituted FΔ*ccdAB* for F to mobilize pkil/ccdB, no escaper Tc’s arose in any of the experimental repetitions (Fig. 2B), which likely can be attributed to the absence of CcdA production by transconjugants co-acquiring FΔ*ccdAB* and pkil/ccdB.

### Conjugative delivery of pkil/ccdB strongly suppresses the growth of the entire recipient population

Although entire transconjugant populations can be eliminated through mobilization of plasmids encoding two toxic elements, the extent of killing of a bacterial population also depends on the efficiency of the conjugation system deployed. The F- and pKM101-encoded T4SSs are among the most efficient of the known conjugation machines, with reported transfer frequencies of ~10^−1^ transconjugants per donor (or recipient) or higher in brief, for example, 1 h, matings (57–59). However, even with a 10^−1^ Tc/R transfer frequency, only ~1 × 10^7^ cells in a starting population of 1 × 10^8^ recipients will acquire the plasmid, leaving ~9 × 10^7^ cells free of the transferred element. Over longer mating periods, subsequent rounds of transfer can expand the Tc/R ratio, but plasmid acquisition and fitness costs impacting the relative growth rates of plasmid-carrying vs plasmid-free populations or other fitness costs associated with plasmid carriage will ultimately dictate whether the plasmid proliferates throughout the entire recipient population (60–63).

**Fig 3:**
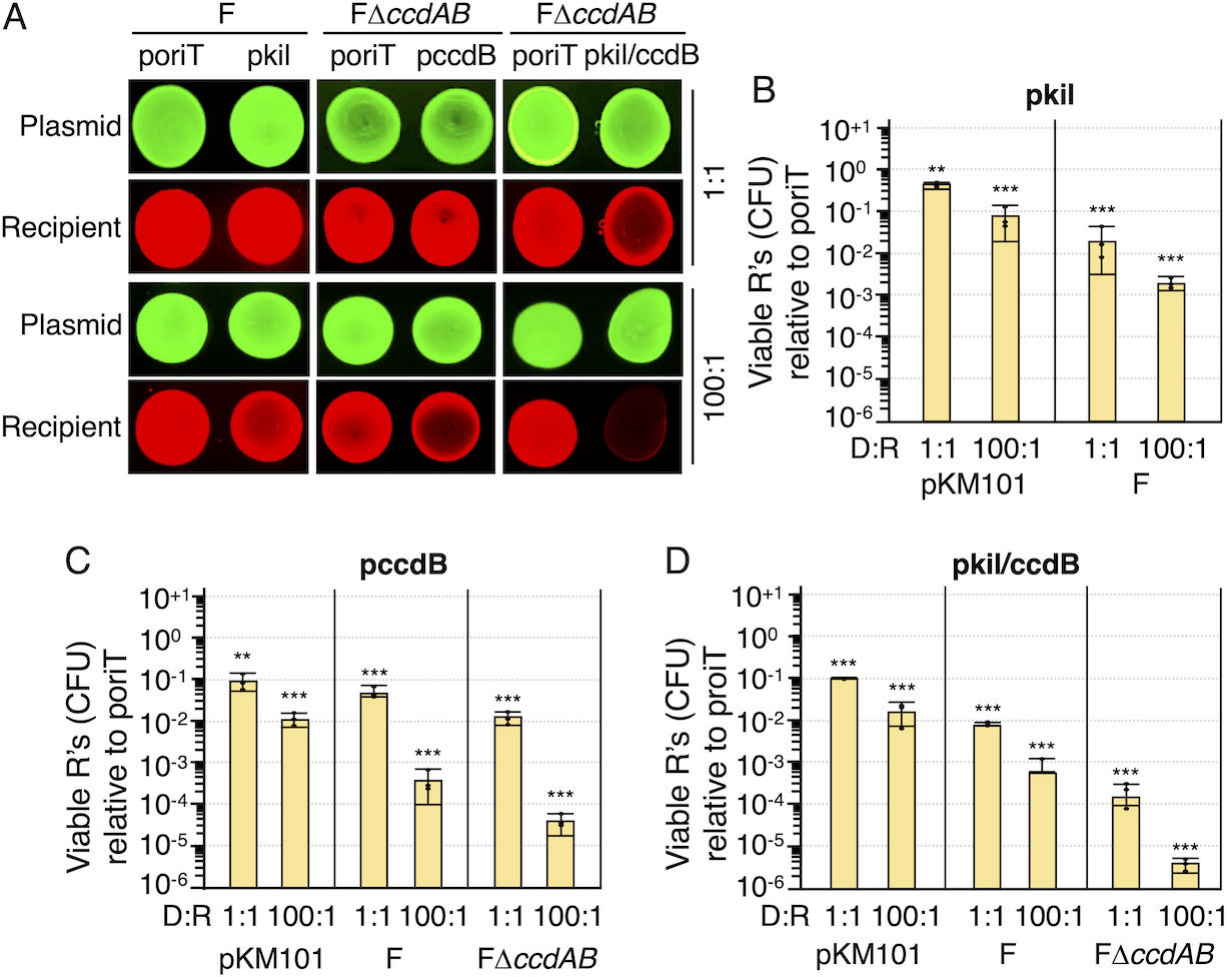
Effects of delivering pkil, pccdB, or pkil/ccdB on the viability of the recipient population. (A) Spot assays. Fluorescently tagged *E. coli* donor (mLemon, green) and recipient (mLychee, red) cells carrying the plasmids indicated at the top were mixed in 1:1 or 100:1 ratios and spotted on LB agar plates. Mating mixes were incubated overnight at 37°C and imaged as described in Materials and Methods. Loss of the mLychee signal indicates growth suppression of the recipient population. (B–D) Viable recipients (R’s; CFUs) resulting from the transfer of (**B**) pkil, (**C**) pccdB, or (**D**) pkil/ccdB relative to transfer of the poriT vector-only plasmid (normalized to 10^0^). Matings were carried out using donor:recipient (D:R) input ratios of 1:1 or 100:1. All matings were repeated at least three times in triplicate, with average recipient killing presented as yellow (2 h solid surface) bars. Recipient CFUs arising from a representative triplicate experiment are shown as black datapoints. Standard deviations are shown as error bars. *P*-values at the top of each bar represent comparisons with the transfer of the poriT control plasmid. **P < 0.001*,* ***P < 0.0001.

To determine how effectively T4SS_pKM_ or T4SS_F_-directed transfer of the killing plasmids suppresses the recipient population, we first introduced a gene encoding the fluorescent reporter mLemon (green/yellow fluorescence) (50) onto the mobilizable poriT vector plasmids, and we expressed mLychee (red fluorescence) (50) in MC4100 recipient cells. Mating mixes composed of donors harboring F (or FΔ*ccdAB*) and recipients were deposited onto LB agar, incubated overnight, and imaged with a ChemiDoc MP Imaging System (see Materials and Methods). Initially, we used a Donor:Recipient (D:R) input ratio of 1:1, and although delivery of pkil or pccdB did not detectably affect recipient cell fluorescence levels, the transfer of pkil/ccdB resulted in a pronounced reduction in red fluorescence. We next used a D:R input ratio of 100:1 and observed that pkil and pccdB transfer conferred slight reductions in recipient cell fluorescence, while transfer of pkil/ccdB elicited a dramatic loss of the recipient signal (Fig. 3A).

The above findings suggested that the delivery of pkil/ccdB suppresses the growth of nearly the entire recipient cell population, especially with a D:R input ratio of 100:1 over a prolonged overnight mating period. We next quantified the effects of conjugation-mediated mobilization of the pkil, pccdB, or pkil/ccdB plasmids in 2 h solid-surface matings. With pKM101 as the delivery system and a D:R ratio of 1:1, donors suppressed the growth of recipient cells at most by 10^1^-fold upon mobilization of pkil, pccdB, or pkil/ccdB (Fig. 3B through D). A D:R ratio of 100:1 suppressed recipient cell viability further, but only by another ~10^1^-fold. F-mediated delivery of the killing plasmids was more effective than pKM101; with a 1:1 D:R ratio, transfer of the killing plasmids elicited 10^1-2^-fold reductions in recipient cell viability, and with a 100:1 ratio conferred >10^3^-fold killing effects (Fig. 3B through D). Most strikingly, donors equipped with FΔ*ccdAB* for mobilization of pccdB or pkil/ccdB conferred even greater killing of the recipient populations than observed for F-directed transfer, with a nearly 10^6^-fold killing effect observed for pkil/ccdB transfer using a D:R of 100:1 (Fig. 3C and D). These findings show that the F system is considerably more effective in killing the recipient cell population than the pKM101 system, in agreement with our earlier findings concerning the killing effects of these respective delivery systems on transconjugant populations (Fig. 1A and B). Also reminiscent of our earlier findings (Fig. 1B and 2B), deployment of donors equipped with FΔ*ccdAB* was significantly more effective than F in growth suppression of the recipient population, presumably because, in contrast to F, the co-transfer of FΔ*ccdAB* with pccdB or pkil/ccdB does not mitigate CcdB toxicity through production of the CcdA antidote.

### Capsule has little effect on pKM101- or F-mediated transfer to or killing of *E. coli* or *Klebsiella pneumoniae* recipients

Bacterial capsule is an essential virulence factor of many pathogens (64–66). Recent experimental studies exploring the role of capsules in horizontal gene transfer showed that *E. coli* donors harboring certain conjugative plasmids deliver those plasmids to capsulated *K. pneumoniae* strains at slightly lower levels (~10^1^- to 10^2^-fold) than to nonencapsulated strains (67, 68). On the other hand, an analysis of thousands of bacterial genomes showed that capsulated bacteria have larger pan-genomes, higher rates of horizontal gene transfer, and higher rates of homologous recombination in their core genomes than nonencapsulated strains, suggestive of a positive role for capsule production in promoting horizontal spread and genetic diversity (69). These findings prompted us to ask whether capsule production impacts the acquisition of the F (IncF) and pKM101 (IncN) plasmids or cell viability upon receipt of toxin-producing plasmids.

**Fig 4:**
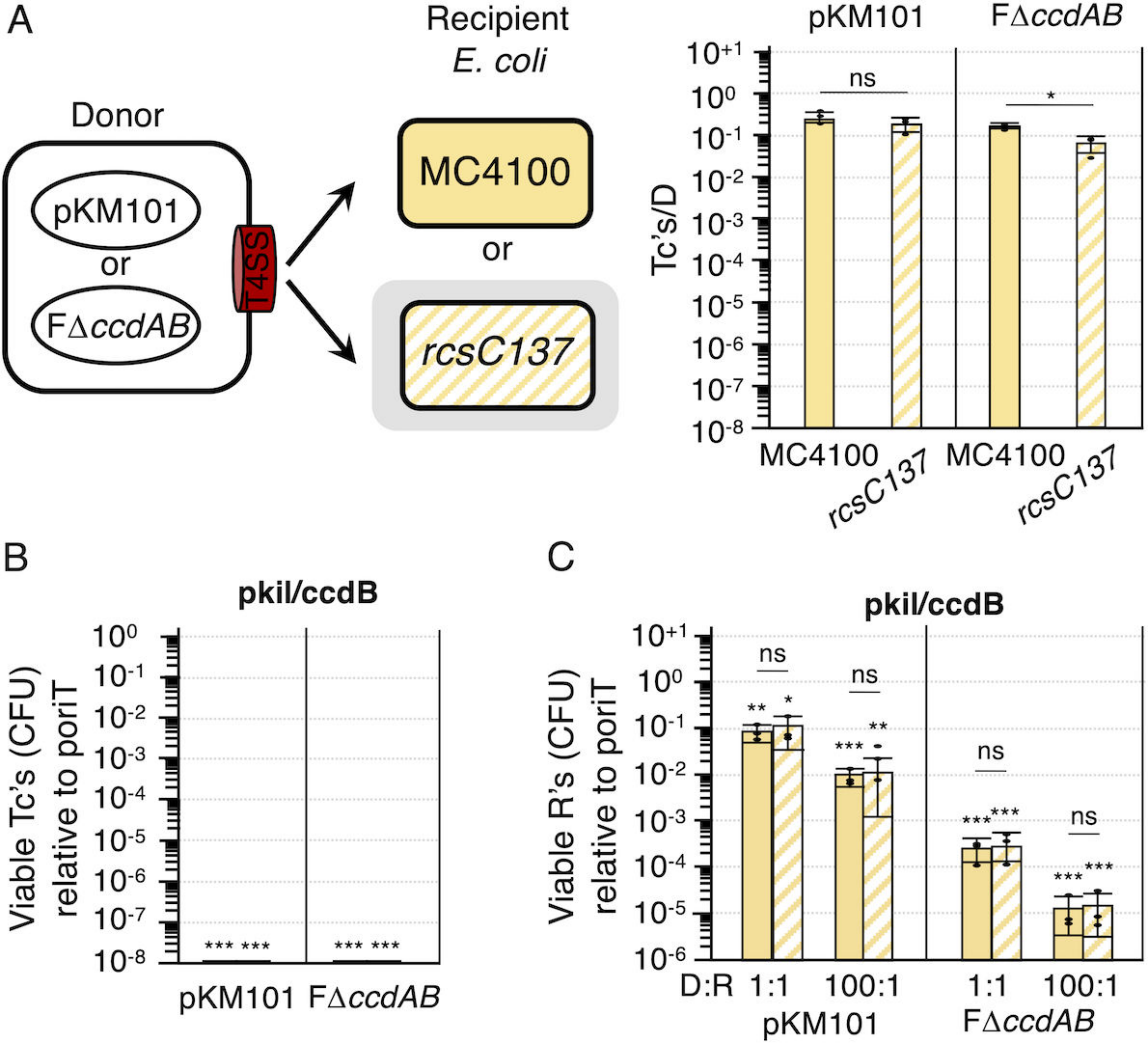
Effects of capsule production by *E. coli* recipients on plasmid acquisition and plasmid-mediated growth suppression. (**A**) Left: Schematic depicting matings between *E. coli* MC4100 donors carrying pKM101 or FΔ*ccdAB* and MC4100 or the isogenic, capsulated variant *rcsC137*. Right: Transconjugants (Tc’s) arising from 2 h solid-surface matings, presented as Tc’s per donor (Tc’s/D). (**B and C**) Effects of pKM101- or FΔ*ccdAB*-mediated mobilization of pkil/ccdB on growth suppression of the (**B**) transconjugant or (**C**) recipient populations. Transconjugant and recipient killing is presented as viable Tc’s or R’s resulting from transfer of pkil/ccdB relative to viable Tc’s or R’s resulting from transfer of cognate poriT plasmids (normalized to 10^0^). All matings were repeated at least three times in triplicate with average transfer frequencies as solid (MC4100) or striped (*rcsC137*) yellow bars. Values arising from a representative triplicate experiment are shown as black datapoints. Standard deviations are shown as error bars. *P*-values at the top of each bar represent comparisons with transfer of the poriT negative control plasmid (normalized to 10^0^). Underlined *P*-values represent comparisons between nonencapsulated and capsulated strains. ***P < 0.0001; **P < 0.001; *P < 0.01; ns, not significant.

In the above mating experiments, we used nonencapsulated *E. coli* strain MC4100 as the recipient. We next compared pKM101 and F plasmid transfer frequencies to MC4100 vs an isogenic mutant *rcsC137*, which produces thick capsule when grown in rich LB media (70). Interestingly, pKM101 transferred at equivalent frequencies to both strains, whereas FΔ*ccdAB* showed only a slight ~0.5 log reduction in transfer to the capsulated variant (Fig. 4A). Although prior studies had not tested the effect of capsule production on pKM101 or F transfer with *E. coli–E. coli* matings, our findings for F acquisition agree with results of a recent study showing that capsulated and nonencapsulated strains of *K. pneumoniae* acquire F plasmids from *E. coli* donors at similar frequencies (68). Mobilization of the pkil/ccdB plasmids by donors carrying pKM101 or FΔ*ccdAB* resulted in killing of the entire transconjugant populations regardless of capsule production (Fig. 4B). pkil/ccdB mobilization also reduced the total recipient CFUs of both capsulated and nonencapsulated strains by similar levels. For the pKM101 system, pkil/ccdB transfer reduced recipient cell viability by 10^1^- to 10^2^-fold using D:R’s of 1:1 and 100:1, and for the FΔ*ccdAB* system, delivery of the killing plasmid conferred reductions of 10^2^- to nearly 10^5^-fold using D:R’s of 1:1 and 100:1, respectively (Fig. 4C).

**Fig 5:**
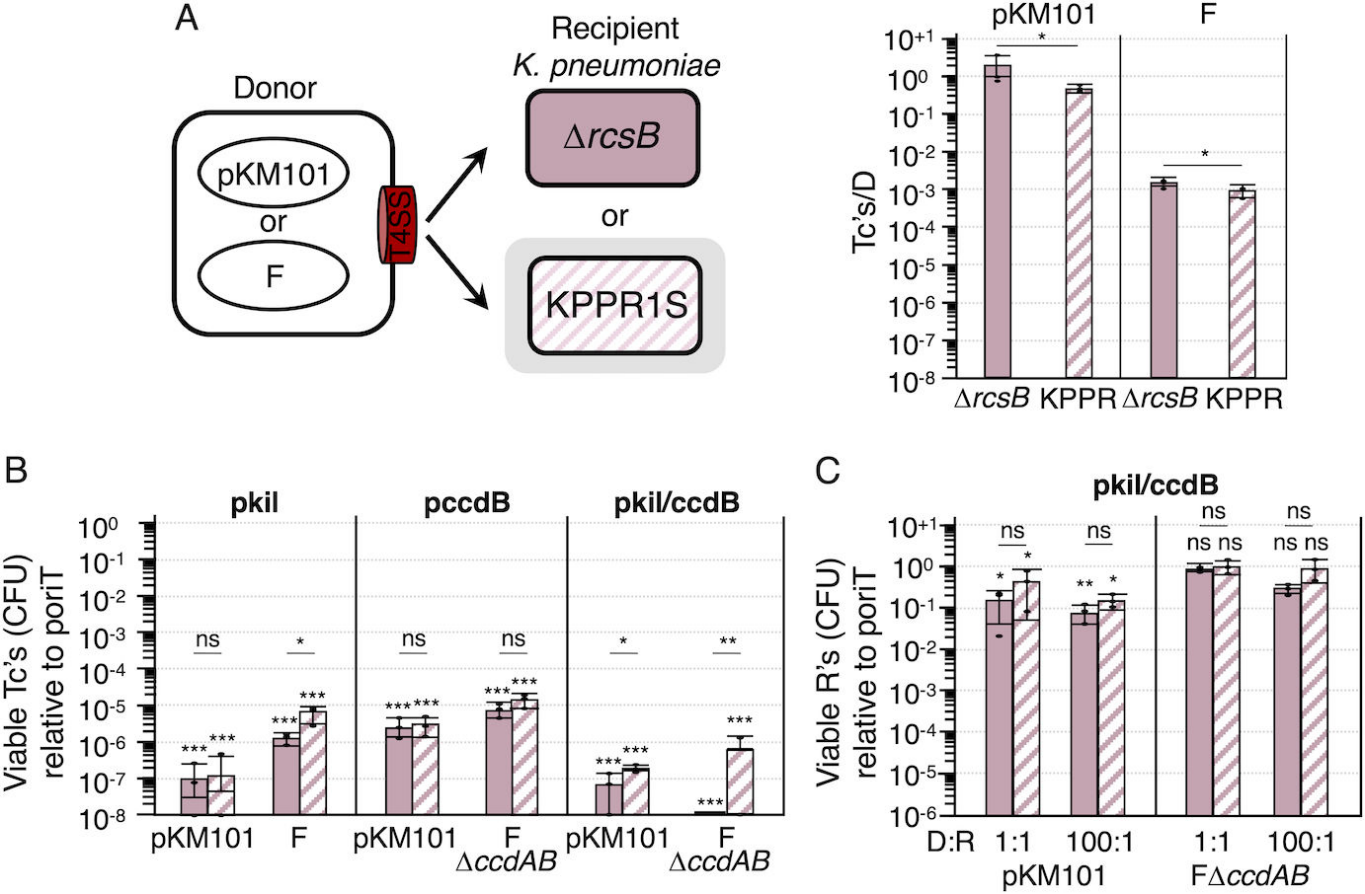
Effects of capsule production by *K. pneumoniae* recipients on plasmid acquisition and plasmid-mediated growth suppression. (**A**) Left: Schematic depicting matings between *E. coli* MC4100 donors carrying pKM101 or F and capsulated KPPR1S and its isogenic, nonencapsulated variant Δ*rcsB*. Right: Transconjugants (Tc’s) arising from 2 h solid-surface matings, presented as Tc’s per donor (Tc’s/D). (**B and C**) Effects of pKM101-, F-, or FΔ*ccdAB*-mediated mobilization of pkil, pccdB, or pkil/ccdB on growth suppression of the (**B**) transconjugant or (**C**) recipient populations. Transconjugant and recipient killing is presented as viable Tc’s or R’s resulting from transfer of the toxin-expressing plasmids relative to viable Tc’s or R’s resulting from transfer of cognate poriT plasmids (normalized to 10^0^). All matings were repeated at least three times in triplicate with average transfer frequencies as solid (Δ*rcsB*) or striped (KPPR1S) pink bars. Values arising from a representative triplicate experiment are shown as black datapoints. Standard deviations are shown as error bars. *P*-values at the top of each bar represent comparisons with the transfer of the poriT control plasmid. Underlined *P-*values represent comparisons between nonencapsulated and capsulated strains. ***P < 0.0001; **P < 0.001; *P < 0.01; ns, not significant.

Next, we tested the effect of capsule production by *K. pneumoniae* on plasmid acquisition and plasmid-mediated toxicity. As recipients, we used the capsulated *K. pneumoniae* strain KPPR1S and the isogenic nonencapsulated variant Δ*rcsB* (41). *E. coli* donors delivered pKM101 or F to capsulated strain KPPR1S at slightly lower, but statistically significant, levels than to the nonencapsulated Δ*rcsB* variant (Fig. 5A). Interestingly, the transfer frequencies of pKM101 approximated those observed with *E. coli–E. coli* matings, but F transferred to *K. pneumoniae* recipients at only modest (10^−3^ Tc’s/D) frequencies compared with *E. coli* recipients (Fig. 4A and 5A). As observed with the *E. coli - E. coli* matings, pKM101- or F-mediated delivery of pkil, pccdB, or pkil/ccdB elicited strong killing of both capsulated and nonencapsulated *K. pneumoniae* Tc’s, with pkil/ccdB conferring the strongest effect on Tc viability (Fig. 5B). In general, capsule production conferred no or slight protective effects on Tc populations from pkil/ccdB-mediated killing.

Despite efficient delivery of pKM101 to *K. pneumoniae* (Fig. 5A) and pKM101-directed growth suppression of the Tc population (Fig. 5B), mobilization of pkil/ccdB had little effect on reducing the total recipient cell populations (Fig. 5C). FΔ*ccdAB*-mediated mobilization of pkil/ccdB failed to suppress growth of the recipient population at detectable levels, but this was not surprising because F itself transferred at modest (10^−3^ Tc’s/D) frequencies to these *K. pneumoniae* strains (Fig. 5C).

Interestingly, pKM101 carries genes for toxins with reported lethal effects on *E. coli* or *Klebsiella* hosts (53, 71, 72). Some genes, designated as *kilA*, *kilB*, or *kilC,* are unrelated to λ-encoded *kil* and kill their hosts only when expressed in the absence of corresponding *kil* override (*kor*) genes. One gene, however, *kikA*, has the unusual property among plasmid- or phage-encoded toxins in its selective toxicity: it kills *Klebsiella* without affecting the viability of *E. coli* hosts (72–74). In fact, the *kikA* locus carries two *orfs*, one embedded within the second, and both Orf products are necessary for *Klebsiella* killing. The *kikA* phenotype manifests as reversible growth inhibition without pronounced effects on cellular respiration or cell morphology (53, 73). Although the mechanism of growth inhibition is not yet known, this narrow-host range activity could be valuable for selective killing of *Klebsiella,* reminiscent of the specificity of CRISPR-Cas9-based killing systems. In the initial studies, *kikA* expression was reported to suppress the growth of *K. pneumoniae*, but the strain used in those studies (M5a1) was subsequently reannotated as *K. oxytoca* (71, 75, 76). To determine whether *kikA* kills *K. pneumoniae* KPPR1S, we again mated pKM101-carrying *E. coli* donors with KPPR1S strains and monitored the growth-suppressive effects of the Tc and R populations. No growth suppression was observed, regardless of KPPR1S capsule production (Fig. S3A). We also cloned *kikA* onto poriT plasmids, but neither pKM101- nor F-mediated delivery of the pkikA plasmids conferred killing of KPPR1S (Fig. S3B), nor did KPPR1S transconjugants that did arise show any growth defects upon induction of *kikA* expression from the P_tac_ promoter (Fig. S3C). In an earlier study, we confirmed that pKM101 encodes an active form of KikA, as evidenced by its requirement for production of the pKM101 pilus and efficient pKM101 transfer in *E. coli–E. coli* matings (58). Taken together with the results of the previous studies, our findings establish that KikA is required for pKM101-encoded T4SS functions in *E. coli* and is toxic to *K. oxytoca* but does not suppress the growth of *K. pneumoniae*.

## DISCUSSION

The conjugative delivery of CRISPR-Cas9 systems elicits several log-fold killing of *E. coli* transconjugant populations, but one limitation of these killing systems is that significant numbers of Tc’s also resist growth suppression. Resistant cells can arise by various mechanisms, including mutational inactivation of the CRISPR-Cas9 elements or target DNA sequences, or repair of CRISPR-induced DSBs by host repair pathways (25). As an alternative to CRISPR-Cas9 elements, conjugative plasmids can be equipped with toxin genes whose expression in targeted cells confers killing. In comparing CRISPR-Cas9- and toxin-based killing strategies, we found that production of the CcdB or Kil toxins elicited appreciably higher levels of killing than our CRISPR-Cas9-based system, especially when killing plasmids were mobilized by the F system. Even so, some transconjugant cells escaped killing, the number varying depending on the conjugation system and killing plasmids deployed. Through co-delivery of CRISPR-Cas9 and a toxin gene or two toxin genes, we essentially eliminated “escaper” transconjugants, and strikingly these combinatorial treatments also elicited a 10^4^-fold or higher growth suppression of the entire *E. coli* recipient populations. Our findings offer conjugation-based killing strategies as a means of reducing bacterial loads at infection sites at a scale potentially sufficient to eliminate the need for additional antibiotic treatment regimens.

In addition to implementing a combinatorial toxin delivery strategy, several other features of our conjugation-based killing system accounted for significant growth suppression of entire recipient cell populations. First, we deployed the highly efficient F and pKM101 systems, both of which can deliver cargoes at frequencies approaching 1 Tc/D or R; such high-frequency-transfer systems maximize the chances that killing plasmids will be disseminated through primary donor-recipient cell contacts. In addition, we also cloned the *oriT* sequences from the conjugative F or pKM101 plasmids onto the killing plasmids. This resulted in co-delivery of the self-transmissible and mobilizable killing plasmids to the same recipients, thus setting the stage for reiterative rounds of secondary transfer and proliferative killing. Finally, we deployed high-copy ColE1 plasmids as the backbones for the construction of the killing plasmids. This yielded high toxin gene dosages and abundant toxin production in new transconjugant cells upon plasmid acquisition and copy number reestablishment. And as an associated practical advantage, constructing small, high-copy ColE1-based plasmids as killing plasmids is considerably simpler and faster than the more laborious recombineering procedures needed for manipulation of large conjugative plasmids.

Our findings expanded on prior reports demonstrating growth-suppressive effects from conjugative transfer of other toxin genes, including genes encoding the ColE3 or ColE7 colicins (77, 78), CcdB interrupted by an intein (79), or the OpaL aggregating antimicrobial peptide (80). However, those killing systems generally relied on the transmission of toxin genes carried on the nonself-transmissible plasmid RSF1010 or large conjugative plasmids such as F or RP4. Although RSF1010 can be mobilized through many different conjugation systems, promiscuous transfer comes at a cost of an appreciably lower transfer frequency (~10^−3^ - 10^−5^ Tc’s/D) than that of the mobilizing plasmid or of the killing plasmids deployed here (81–83). On the other hand, whereas F and other self-transmissible plasmids equipped with toxin genes can rapidly spread the toxic element through the recipient population, this comes at a cost of a low toxin gene dosage due to the low copy numbers (~1–2) of these large plasmids (19, 84).

Reminiscent of previous findings (79), we determined that conjugative transfer of *ccdB* was highly effective in growth suppression, especially when F was deleted of the *ccdA* antitoxin co-transferred with the pccdB killing plasmid. Importantly, we also showed that constitutive chromosomal expression of *ccdA* abolished CcdB toxicity in donor cells, which allows *ccdB*-mediated killing without the need for incorporating an intein to control CcdB toxicity. We also demonstrated efficient killing of target cells by λ phage-encoded Kil. Kil is a member of a large and functionally diverse family of phage-encoded antimicrobial peptides (AMPs) that have received considerable attention in recent years for their therapeutic potential as alternatives or adjuncts to antibiotic treatments (85–87). While many AMPs exert their effects by disrupting bacterial cell envelope integrity, λ Kil acts from within the cell by associating with the inner membrane, where it induces filamentation and killing of *E. coli* cells through rapid inhibition of FtsZ ring formation in a ZipA-dependent manner (36, 37, 56). In principle, other AMPS, or indeed any other antibacterial toxin capable of suppressing the growth of any Gram-negative or -positive species, are viable elements for incorporation into conjugation-based killing systems. As in the case of CcdB or Kil, certain toxic elements block conserved pathways present in many bacteria and thus should have broad-spectrum activities, whereas others, such as KikA or CRISPR systems, may block specific pathways or target specific DNA sequences for restricted killing only of a given species. Given that most or all bacterial species are susceptible to acquisition of foreign DNA by conjugation, we further predict that any bacterial species of interest is a viable target for killing, provided that judicious choices are made concerning the conjugation system, the killing plasmid backbone, and the toxic elements for assembly of the killing system.

Despite the broad potential of conjugation-based therapies, various physiological or environmental conditions can influence killing efficiencies. Here, we determined that one physiological state, the capacity to produce a thick extracellular capsule, had little effect on the acquisition of the pKM101 or F plasmids by *E. coli* or *K. pneumoniae* recipients. Moreover, mobilization of the pkil/ccdB killing plasmid elicited similar levels of growth suppression of the isogenic capsulated and nonencapsulated *E. coli* strains. We did, however, observe that *K. pneumoniae* recipients acquired the classical F plasmid at ≥10^2^-fold lower frequencies than *E. coli* recipients, regardless of capsule production. We suspect this can be attributed to a lack of productive binding between the F-encoded TraN surface protein and KPPR1S-encoded OMPs, as such interactions have recently been shown to influence F plasmid transfer rates and species host range (30, 31). Various F plasmids naturally reside in and efficiently transfer between *K. pneumoniae* strains, for example, pKpQil (88), and it will be interesting in future studies to assess whether such plasmids could be used for more efficient targeted killing of *K. pneumoniae* recipients. We also found that appreciably higher numbers of *K. pneumoniae* Tc escaper cells arose upon acquisition of pkil/ccdB plasmids than detected, with the corresponding *E. coli–E. coli* matings. This could indicate that *K. pneumoniae* is intrinsically more resistant to the CcdB and Kil toxins than *E. coli*; if so, further studies exploring the efficacy of other toxic elements for *K. pneumoniae* killing are warranted. We also detected statistically significantly higher numbers of viable Tc’s among capsulated KPPR1S compared with nonencapsulated Δ*rcsB* transconjugants upon acquisition of the pkil or pkil/ccdB plasmids. Capsule production thus might afford some protection at least against Kil toxicity in this species. Finally, again in contrast to the situation with *E. coli–E. coli* matings, we found that neither pKM101- nor F-mediated transfer of pkil/ccdB conferred appreciable killing of the *K. pneumoniae* recipient populations regardless of capsule production or, intriguingly, the D:R input ratio. These findings might be attributable to intrinsic limitations in the spread of these plasmids among the *K. pneumoniae* recipient population, reinforcing the need for further empirical studies aimed at optimizing killing systems intended for *K. pneumoniae* growth suppression.

Other potential limitations must be confronted for widespread deployment of conjugation-based targeted killing in clinical or environmental settings. For example, our findings underscore the need to deploy highly efficient conjugation systems, ideally capable of delivering DNA cargoes at frequencies approaching 1 Tc/D or R in short-term matings. Such high-efficiency conjugation systems exist among the Gram-negative Enterobacteriaceae, for example, F, pKM101, RP4, and certain Gram-positive species, for example, *Enterococcus faecalis* pCF10, but they are clearly not ubiquitous among all bacterial species (89–91). Various strategies can be used to enhance transfer efficiencies of conjugation systems, including the isolation of mutations in regulatory loci or components of the transfer apparatus that yield elevated transfer rates. In addition, recent studies have demonstrated the capacity of programmed delivery systems (PDSs) to significantly enhance targeted DNA transfer events to a given species. Engineering of these PDSs involves the introduction of nanobodies (Nbs) to the cell surfaces of donor cells with binding specificities for antigens (Ags) naturally present on the surfaces of targeted cells (18, 92). Nb-Ag bridges can elevate transfer frequencies by 10^1^- to 10^3^-fold to a target species of interest even under conditions of low cell density and polymicrobial growth (18, 92).

Regardless of efforts to enhance conjugation efficiencies, productive encounters can be limited by the structural organization of donor and recipient cells, a factor complicated by cell division events that can physically prevent donors from accessing new recipient cell progeny. A recent elegant study highlighted the importance not only of donor-recipient encounter rates but also of the engagement time (defined as the interval required between two successful matings) as critical limiting factors for conjugation (60). In nature, even more complicated microbial community structures, other physical barriers, the physiological state of donors and recipients, and various environmental influences affecting gene expression patterns can further impede conjugative dissemination of killing plasmids throughout target cell populations. The extent to which conjugation-based killing strategies can be developed to overcome intrinsic limitations in recipient cell access for saturated cargo delivery remains an important question for future studies.

Finally, to implement killing systems for disease treatments, donor cells must be capable of penetrating eukaryotic host tissues with minimal impact on host immune or inflammatory responses. To this end, *E. coli* donors with cell envelope mutations or probiotic strains such as Nissle 1917 are currently the best options to achieve these ends (7, 93). Recently, we showed that anucleate minicells, which are the nonviable but metabolically active products of asymmetric cell division, are highly proficient for conjugative delivery of DNA cargoes to intact cells. When derived from Nb-displaying cells, minicells also preferentially and abundantly bind to antigen-presenting target cells (18). In view of these findings, it is enticing to propose that minicells derived from probiotic strains, and equipped with Nb’s and the killing systems described herein, constitute a promising infection treatment strategy with minimal negative host responses.

## SUPPLEMENTAL MATERIAL

Supplemental figures jb.00168-25-s0001.pdf Fig. S1 to S3.

ASM does not own the copyrights to Supplemental Material that may be linked to, or accessed through, an article. The authors have granted ASM a non-exclusive, world-wide license to publish the Supplemental Material files. Please contact the corresponding author directly for reuse.
