# Supplementary material for: Conjugative delivery of toxin genes ccdB and kil confers synergistic killing of bacterial recipients
Source: J Bacteriol. 2025 Jul 3;207(7):e00168-25. doi: 10.1128/jb.00168-25 (PMC12288468; doi:10.1128/jb.00168-25)
Supplement: Supplemental figures — Fig. S1 to S3. [file jb.00168-25-s0001.pdf]

Supplemental Material for:

Conjugative Delivery of Toxin Genes *ccdB* and *kil* Confers Synergistic Killing of Bacterial Recipients

Yang Grace Li, Daniel Haeusser, William Margolin, and Peter J. Christie

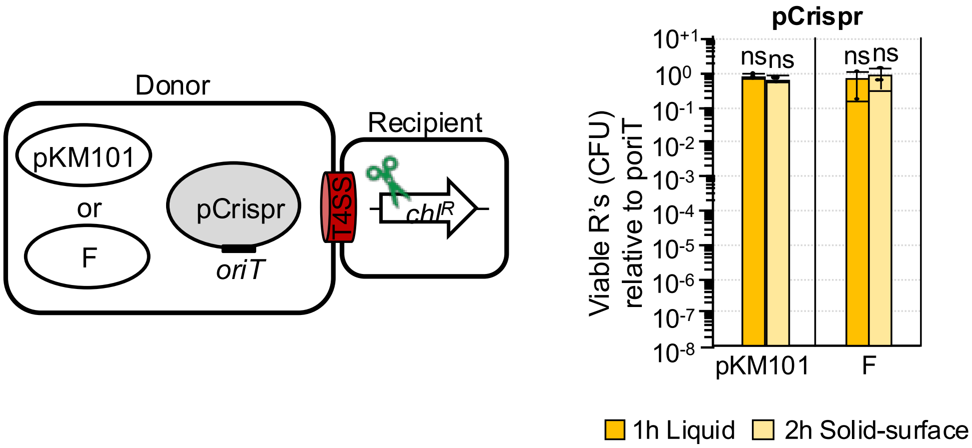

Figure S1. Effects of conjugative transfer of the pCrispr plasmid on recipient viability. **Left:** Schematic depicting matings between *E. coli* MC4100 donors carrying conjugative plasmids and mobilizable pCrispr and recipients with the CRISPR-Cas9 target (*chl*^{R}, scissors). **Right:** Recipient (R) killing presented as viable R's (colony-forming units; CFUs) resulting from the transfer of pCrispr relative to the poriT vector-only plasmid (normalized to 10^0). All matings were repeated at least three times in triplicate with average recipient killing presented as orange (1-h liquid mating) or yellow (2-h solid-surface) bars. Recipient CFU's arising from a representative triplicate experiment are shown as black datapoints. Standard deviations are shown as error bars. *P*-values at the top of each bar represent comparisons with transfer of the poriT negative control plasmid. ns, not significant.

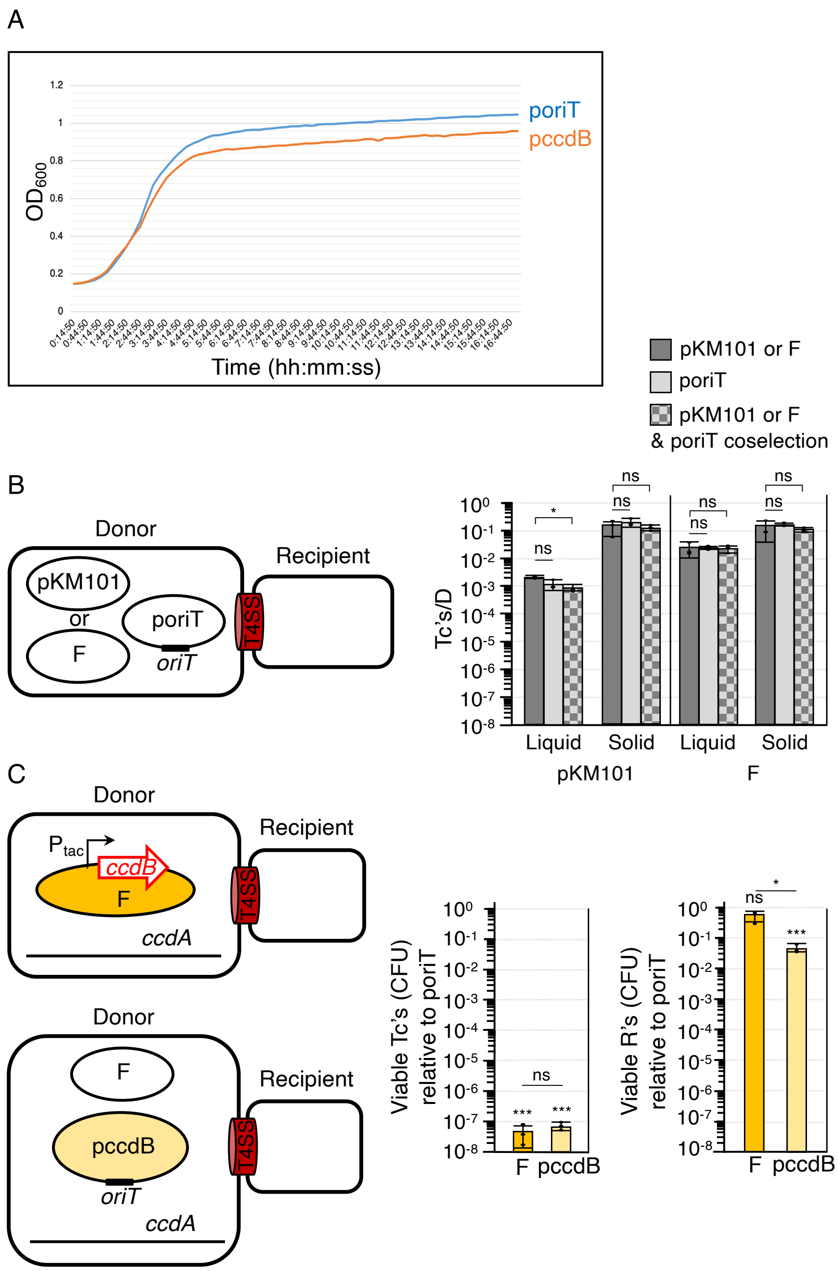

Figure S2. Plasmid co-transfer and killing frequencies. **A)** Growth curves of YGLS9(poriT) and YGLS9(pccdB) generated as described in Materials and Methods. YGSL9 is MC4100 with chromosomally-integrated, constitutively-expressed P_{chl}::*ccdA*. poriT and pccdB carry F plasmid *oriT* sequences. **B)** Left: Schematic depicting matings between *E. coli* MC4100 donors carrying pKM101 or F and the cognate poriT plasmids with plasmid-free recipient MC4100-Rif. Right: Transfer frequencies with transconjugants (Tc's) arising from selection for self-transmissible pKM101 or F alone, the cognate mobilizable poriT plasmid alone, or both self-transmissible and cognate mobilizable plasmids. Tc's arising from 1 h liquid or 2 h solid-surface matings, presented as Tc's per donor (Tc's/D). *P* values represent comparisons between transfer frequencies of pKM101 or F vs cognate poriT plasmids or vs both self-transmissible and cognate mobilizable plasmids. *$P<0.01$; ns, not significant. **C)** Left: Upper schematic depicts *E. coli* YGLS9 donors with F expressing P_{tac}::*ccdB* and chromosomally-integrated P_{chl}::*ccdA* mated with plasmid-free MC4100-Rif. Lower schematic depicts YGLS9 donors carrying F and mobilizable pccdB and chromosomally-integrated P_{chl}::*ccdA* mated with MC4100-Rif. Right: Orange bars correspond to Tc's and recipients (R's) per donor arising from transfer of F harboring P_{tac}::*ccdB*. Yellow bars correspond to Tc's and R's per donor arising from F-mediated transfer of pccdB. Matings were carried out for 2 h on a solid surface. *P*-values at the top of each bar represent comparisons with transfer frequencies of the poriT control plasmid. Underlined *P*-values represent comparisons between Tc or R killing frequencies resulting from acquisition of F carrying P_{tac}::*ccdB* vs pccdB. ***$P<0.0001$; * $P<0.01$; ns, not significant. Panels **B** and **C)** 2 h solid-surface matings were repeated at least three times in triplicate with average transconjugant or recipient killing presented. Tc or R CFU's arising from a representative triplicate experiment are shown as black datapoints. Standard deviations are shown as error bars.

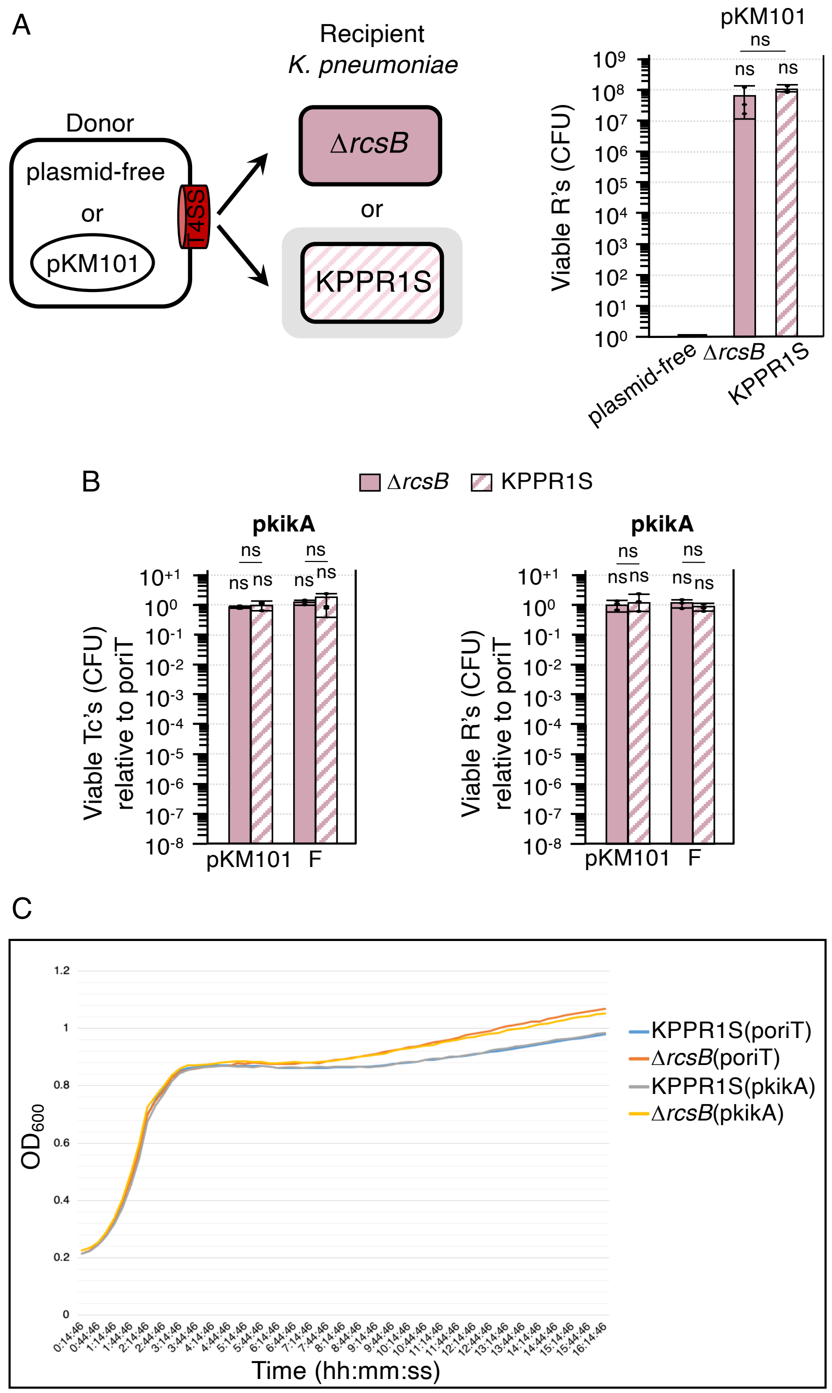

Figure S3. Effects of pKM101-encoded KikA toxin on growth of *K. pneumoniae* KPPR1S strains. **A)** Left: Schematic depicting matings between MC4100 plasmid-free mock donor or MC4100 donor carrying pKM101 and the recipient strains KPPR1S (capsulated) or isogenic variant Δ*rcsB* (nonencapsulated). Right: Recipients (R's) arising from 2-h solid-surface matings, presented as viable R's (CFUs) relative to plasmid-free matings (normalized to 10^0). **B)** Left: Transconjugants (Tc's) arising from 2-h solid-surface matings, presented as viable Tc's (CFUs) resulting from the transfer of pkikA relative to poriT (normalized to 10^0) to KPPR1S or Δ*rcsB* recipients. Right: R's arising from 2-h solid-surface matings, presented as viable R's (CFUs) resulting from the transfer of pkikA relative to poriT (normalized to 10^0) to KPPR1S or Δ*rcsB* recipients. *P*-values at the top of each bar represent comparisons with transfer frequencies of the poriT control plasmid. Underlined *P*-values represent comparisons between nonencapsulated Δ*rcsB* vs encapsulated KPPR1S strains. ns, not significant. All matings were repeated at least three times in triplicate with average transconjugant or recipient killing presented. Tc or R CFU's arising from a representative triplicate experiment are shown as black datapoints. Standard deviations are shown as error bars. **C)** Growth curves of KPPR1S(poriT), KPPR1S(pkikA), Δ*rcsB*(poriT), and Δ*rcsB*(pkikA) generated as described in Materials and Methods. poriT and pkikA carry pKM101 *oriT* sequence.
